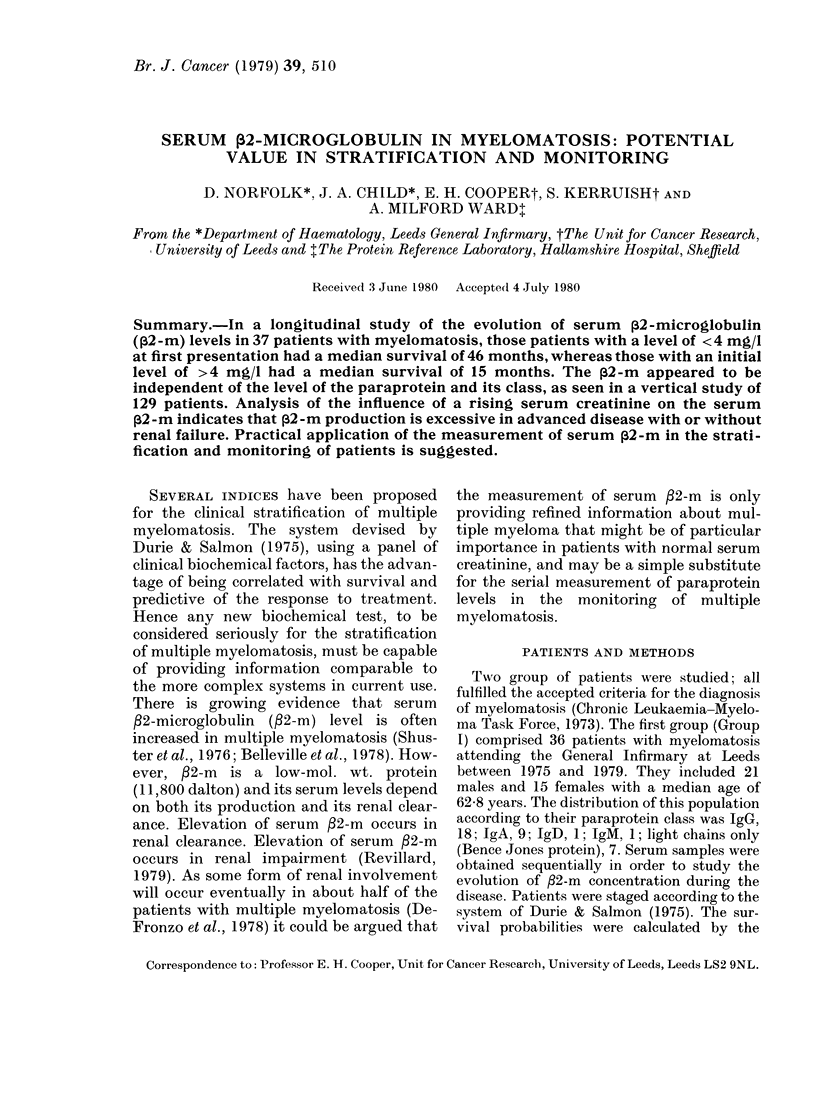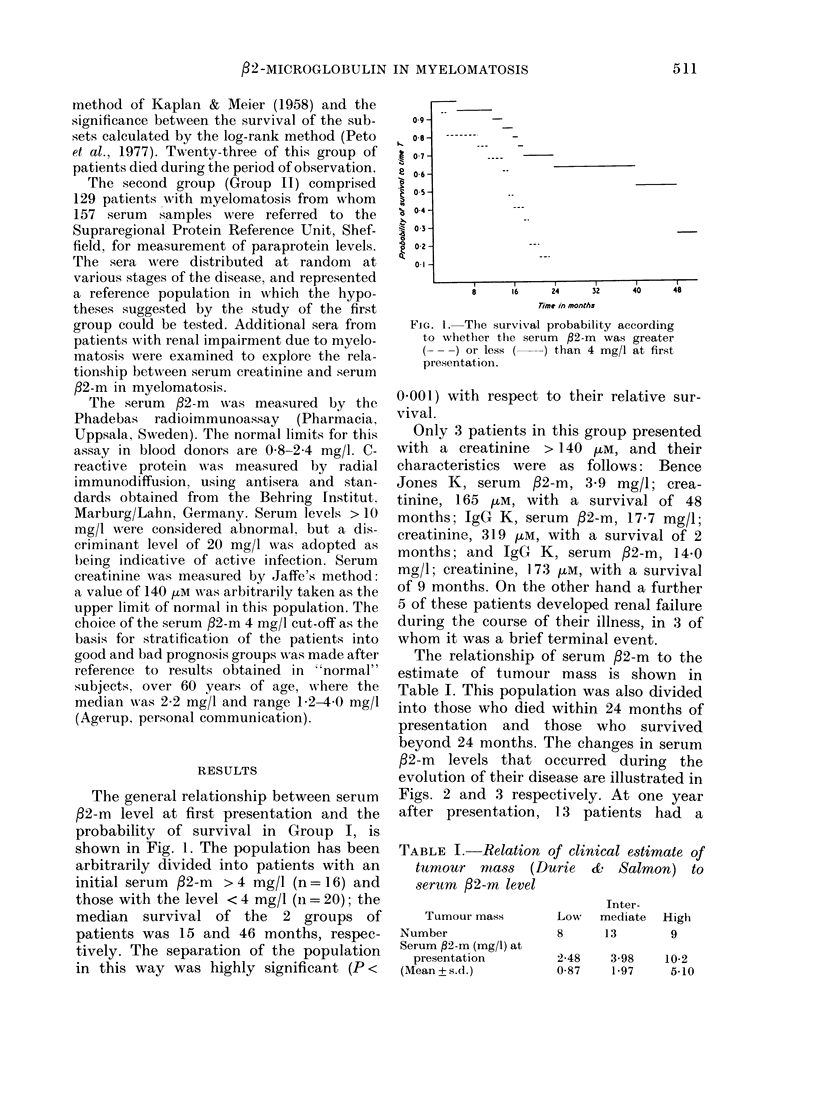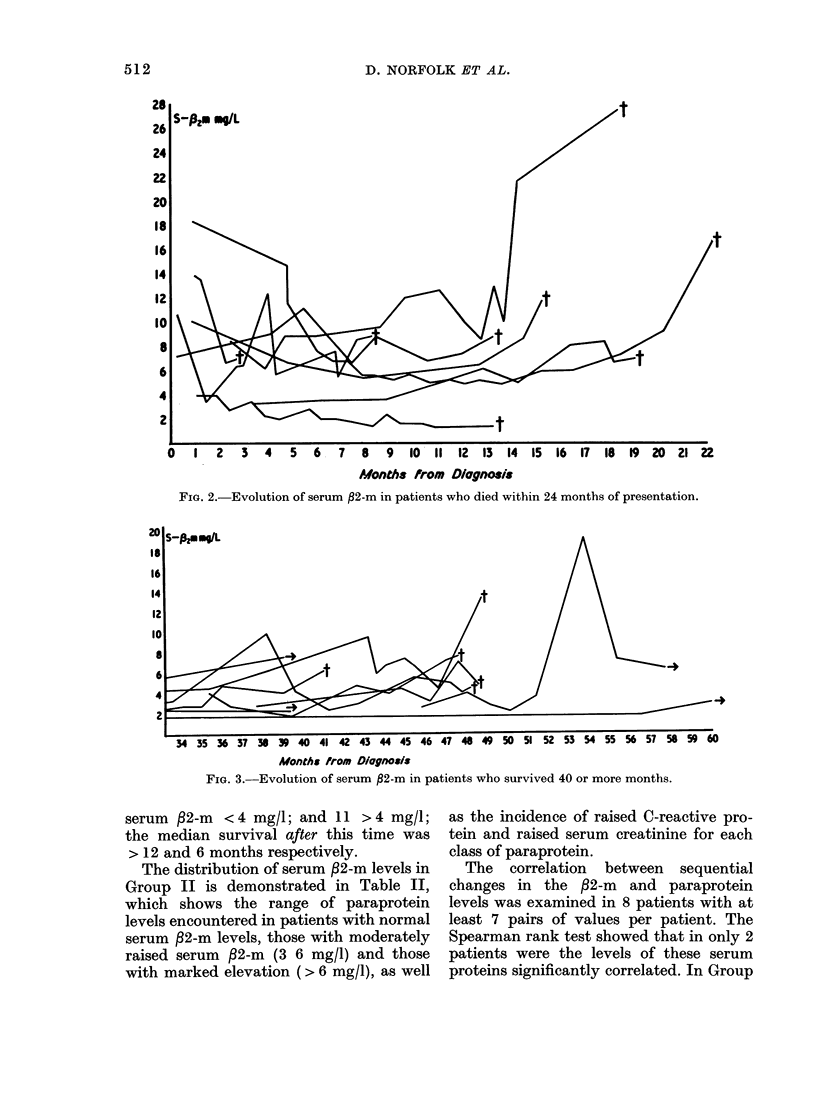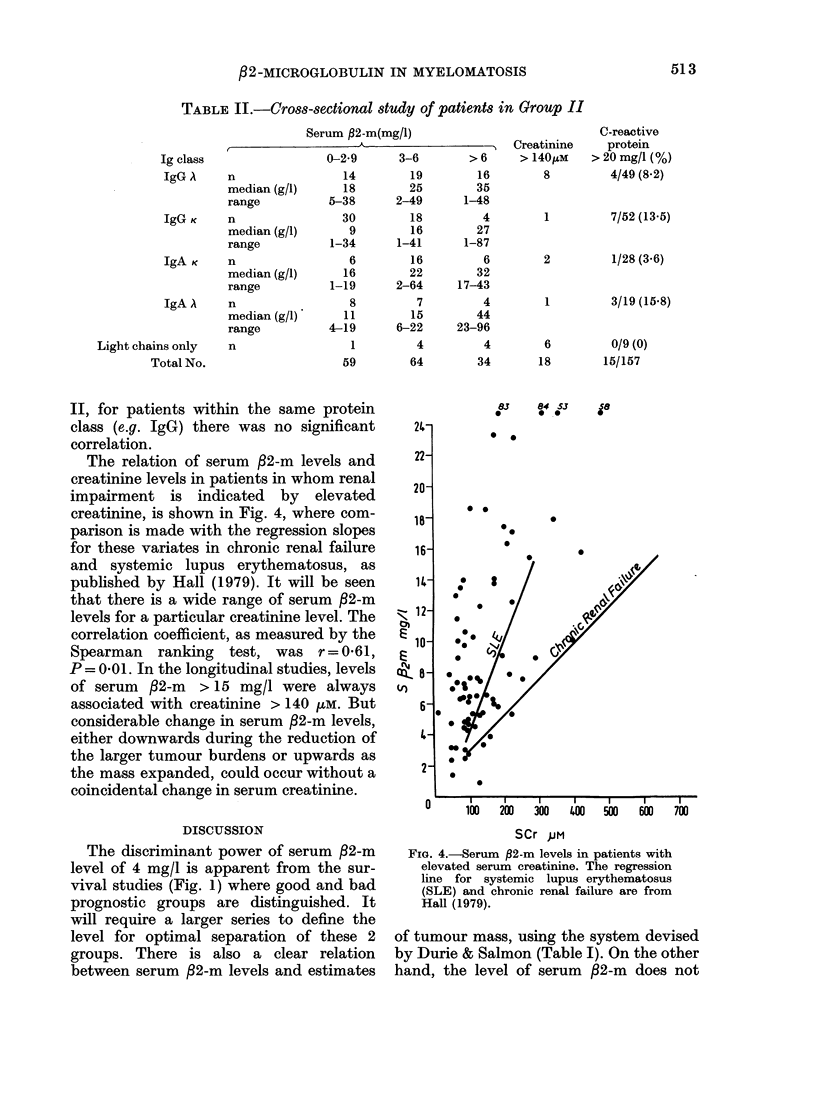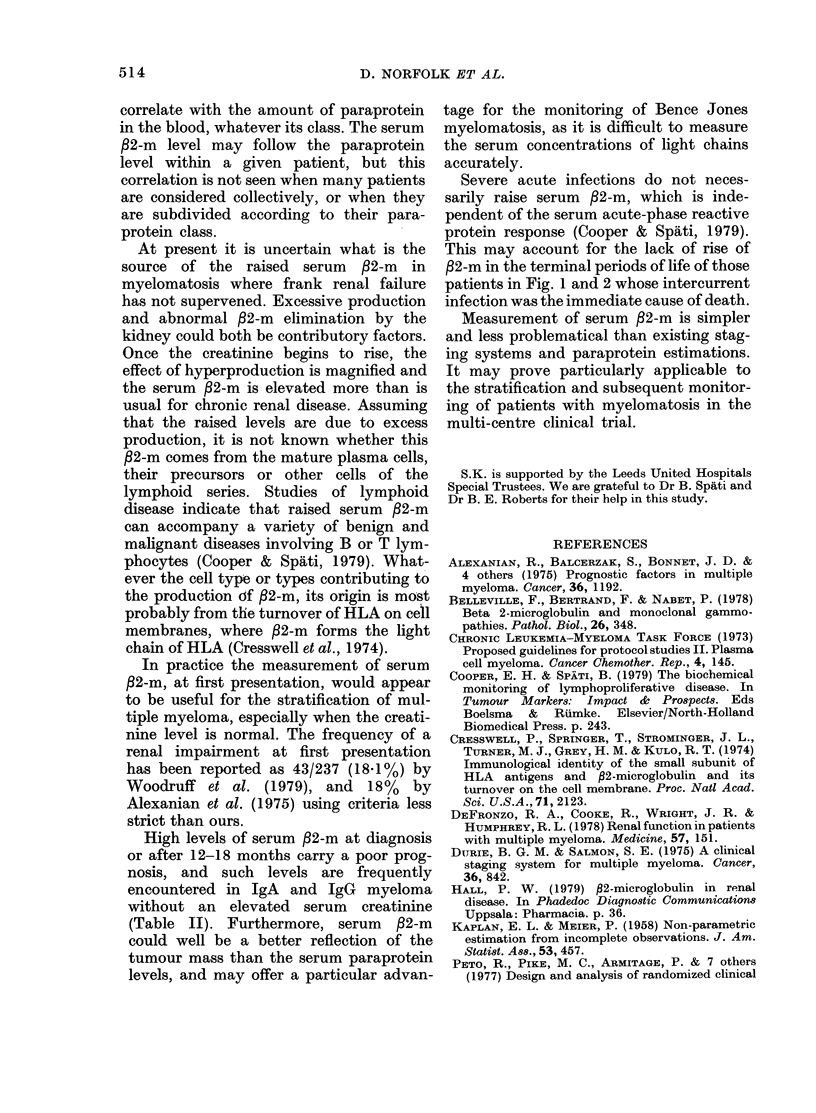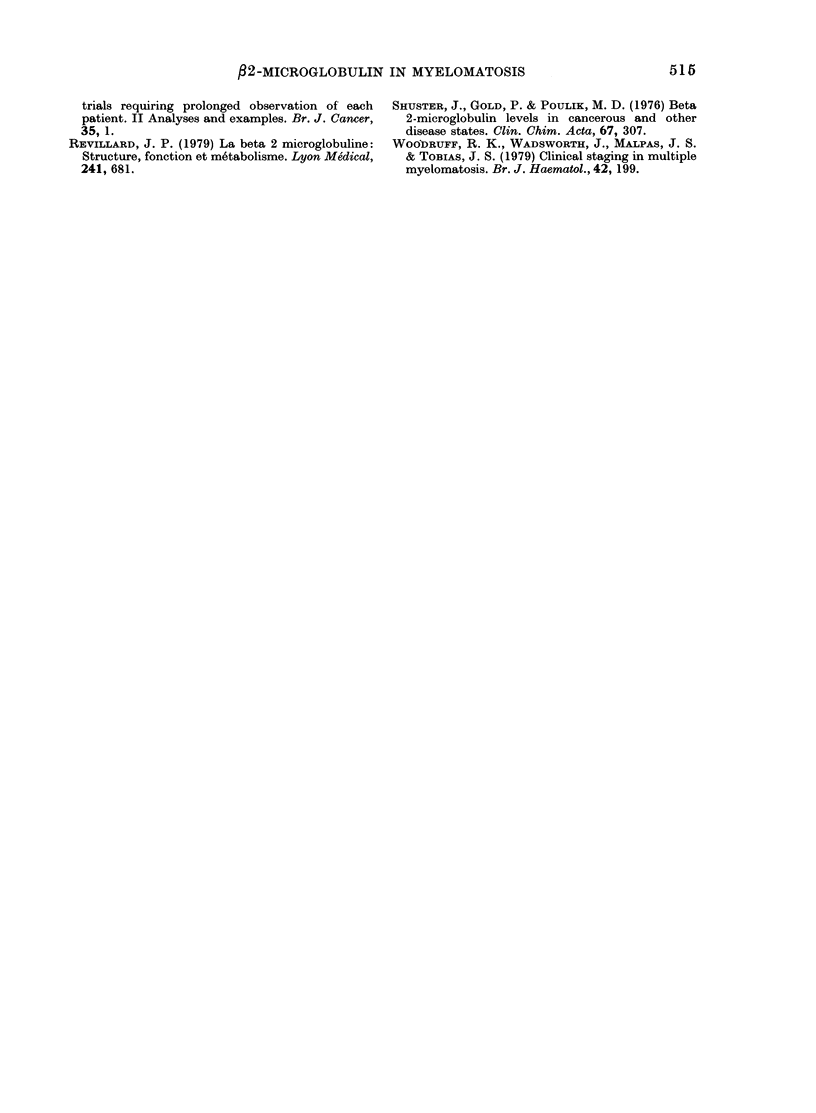# Serum beta 2-microglobulin in myelomatosis: potential value in stratification and monitoring.

**DOI:** 10.1038/bjc.1980.273

**Published:** 1980-10

**Authors:** D. Norfolk, J. A. Child, E. H. Cooper, S. Kerruish, A. M. Ward

## Abstract

In a longitudinal study of the evolution of serum beta 2-microglobulin (beta 2-m) levels in 37 patients with myelomatosis, those patients with a level of < 4 mg/l at first presentation had a median survival of 46 months, whereas those with an initial level of > mg/l had a median survival of 15 months. THe beta 2-m appeared to be independent of the level of the paraprotein and its class, as seen in a vertical study of 129 patients. Analysis of the influence of a rising serum creatinine on the serum beta 2-m indicates that beta 2-m production is excessive in advanced disease with or without renal failure. Practical application of the measurement of serum beta 2-m in the stratification and monitoring of patients is suggested.


*Br. J. Cancer* (1979) **39**, 510

### SERUM β2-MICROGLOBULIN IN MYELOMATOSIS: POTENTIAL VALUE IN STRATIFICATION AND MONITORING

D. NORFOLK*, J. A. CHILD*, E. H. COOPER†, S. KERRUISH† AND A. MILFORD WARD‡

*From the *Department of Haematology, Leeds General Infirmary, †The Unit for Cancer Research, University of Leeds and ‡The Protein Reference Laboratory, Hallamshire Hospital, Sheffield*

Received 3 June 1980 Accepted 4 July 1980

**Summary.—In a longitudinal study of the evolution of serum β2-microglobulin (β2-m) levels in 37 patients with myelomatosis, those patients with a level of <4 mg/l at first presentation had a median survival of 46 months, whereas those with an initial level of >4 mg/l had a median survival of 15 months. The β2-m appeared to be independent of the level of the paraprotein and its class, as seen in a vertical study of 129 patients. Analysis of the influence of a rising serum creatinine on the serum β2-m indicates that β2-m production is excessive in advanced disease with or without renal failure. Practical application of the measurement of serum β2-m in the stratification and monitoring of patients is suggested.**

SEVERAL INDICES have been proposed for the clinical stratification of multiple myelomatosis. The system devised by Durie & Salmon (1975), using a panel of clinical biochemical factors, has the advantage of being correlated with survival and predictive of the response to treatment. Hence any new biochemical test, to be considered seriously for the stratification of multiple myelomatosis, must be capable of providing information comparable to the more complex systems in current use. There is growing evidence that serum β2-microglobulin (β2-m) level is often increased in multiple myelomatosis (Shuster *et al.*, 1976; Belleville *et al.*, 1978). However, β2-m is a low-mol. wt. protein (11,800 dalton) and its serum levels depend on both its production and its renal clearance. Elevation of serum β2-m occurs in renal clearance. Elevation of serum β2-m occurs in renal impairment (Revillard, 1979). As some form of renal involvement will occur eventually in about half of the patients with multiple myelomatosis (DeFronzo *et al.*, 1978) it could be argued that the measurement of serum β2-m is only providing refined information about multiple myeloma that might be of particular importance in patients with normal serum creatinine, and may be a simple substitute for the serial measurement of paraprotein levels in the monitoring of multiple myelomatosis.

### PATIENTS AND METHODS

Two group of patients were studied; all fulfilled the accepted criteria for the diagnosis of myelomatosis (Chronic Leukaemia–Myeloma Task Force, 1973). The first group (Group I) comprised 36 patients with myelomatosis attending the General Infirmary at Leeds between 1975 and 1979. They included 21 males and 15 females with a median age of 62·8 years. The distribution of this population according to their paraprotein class was IgG, 18; IgA, 9; IgD, 1; IgM, 1; light chains only (Bence Jones protein), 7. Serum samples were obtained sequentially in order to study the evolution of β2-m concentration during the disease. Patients were staged according to the system of Durie & Salmon (1975). The survival probabilities were calculated by the

Correspondence to: Professor E. H. Cooper, Unit for Cancer Research, University of Leeds, Leeds LS2 9NL.

method of Kaplan & Meier (1958) and the significance between the survival of the subsets calculated by the log-rank method (Peto *et al.*, 1977). Twenty-three of this group of patients died during the period of observation.

The second group (Group II) comprised 129 patients with myelomatosis from whom 157 serum samples were referred to the Supraregional Protein Reference Unit, Sheffield, for measurement of paraprotein levels. The sera were distributed at random at various stages of the disease, and represented a reference population in which the hypotheses suggested by the study of the first group could be tested. Additional sera from patients with renal impairment due to myelomatosis were examined to explore the relationship between serum creatinine and serum β2-m in myelomatosis.

The serum β2-m was measured by the Phadebas radioimmunoassay (Pharmacia, Uppsala, Sweden). The normal limits for this assay in blood donors are 0·8–2·4 mg/l. C-reactive protein was measured by radial immunodiffusion, using antisera and standards obtained from the Behring Institut, Marburg/Lahn, Germany. Serum levels >10 mg/l were considered abnormal, but a discriminant level of 20 mg/l was adopted as being indicative of active infection. Serum creatinine was measured by Jaffe's method: a value of 140 μM was arbitrarily taken as the upper limit of normal in this population. The choice of the serum β2-m 4 mg/l cut-off as the basis for stratification of the patients into good and bad prognosis groups was made after reference to results obtained in "normal" subjects, over 60 years of age, where the median was 2·2 mg/l and range 1·2–4·0 mg/l (Agerup, personal communication).

### RESULTS

The general relationship between serum β2-m level at first presentation and the probability of survival in Group I, is shown in Fig. 1. The population has been arbitrarily divided into patients with an initial serum β2-m >4 mg/l (n = 16) and those with the level <4 mg/l (n = 20); the median survival of the 2 groups of patients was 15 and 46 months, respectively. The separation of the population in this way was highly significant ($P <$ 0·001) with respect to their relative survival.

FIG. 1.—The survival probability according to whether the serum β2-m was greater (– – –) or less (——) than 4 mg/l at first presentation.

Only 3 patients in this group presented with a creatinine >140 μM, and their characteristics were as follows: Bence Jones K, serum β2-m, 3·9 mg/l; creatinine, 165 μM, with a survival of 48 months; IgG K, serum β2-m, 17·7 mg/l; creatinine, 319 μM, with a survival of 2 months; and IgG K, serum β2-m, 14·0 mg/l; creatinine, 173 μM, with a survival of 9 months. On the other hand a further 5 of these patients developed renal failure during the course of their illness, in 3 of whom it was a brief terminal event.

The relationship of serum β2-m to the estimate of tumour mass is shown in Table I. This population was also divided into those who died within 24 months of presentation and those who survived beyond 24 months. The changes in serum β2-m levels that occurred during the evolution of their disease are illustrated in Figs. 2 and 3 respectively. At one year after presentation, 13 patients had a

TABLE I.—*Relation of clinical estimate of tumour mass (Durie & Salmon) to serum β2-m level*

| Tumour mass | Low | Inter-mediate | High |
|---|---|---|---|
| Number | 8 | 13 | 9 |
| Serum β2-m (mg/l) at presentation | 2·48 | 3·98 | 10·2 |
| (Mean ± s.d.) | 0·87 | 1·97 | 5·10 |

FIG. 2.—Evolution of serum $\beta$2-m in patients who died within 24 months of presentation.

FIG. 3.—Evolution of serum $\beta$2-m in patients who survived 40 or more months.

serum $\beta$2-m $<4$ mg/l; and 11 $>4$ mg/l; the median survival *after* this time was $>12$ and 6 months respectively.

The distribution of serum $\beta$2-m levels in Group II is demonstrated in Table II, which shows the range of paraprotein levels encountered in patients with normal serum $\beta$2-m levels, those with moderately raised serum $\beta$2-m (3 6 mg/l) and those with marked elevation ($>6$ mg/l), as well as the incidence of raised C-reactive protein and raised serum creatinine for each class of paraprotein.

The correlation between sequential changes in the $\beta$2-m and paraprotein levels was examined in 8 patients with at least 7 pairs of values per patient. The Spearman rank test showed that in only 2 patients were the levels of these serum proteins significantly correlated. In Group

TABLE II.—*Cross-sectional study of patients in Group II*

| Ig class | | Serum β2-m(mg/l) 0–2·9 | 3–6 | >6 | Creatinine >140μM | C-reactive protein >20 mg/l (%) |
|---|---|---|---|---|---|---|
| IgG λ | n | 14 | 19 | 16 | 8 | 4/49 (8·2) |
| | median (g/l) | 18 | 25 | 35 | | |
| | range | 5–38 | 2–49 | 1–48 | | |
| IgG κ | n | 30 | 18 | 4 | 1 | 7/52 (13·5) |
| | median (g/l) | 9 | 16 | 27 | | |
| | range | 1–34 | 1–41 | 1–87 | | |
| IgA κ | n | 6 | 16 | 6 | 2 | 1/28 (3·6) |
| | median (g/l) | 16 | 22 | 32 | | |
| | range | 1–19 | 2–64 | 17–43 | | |
| IgA λ | n | 8 | 7 | 4 | 1 | 3/19 (15·8) |
| | median (g/l) | 11 | 15 | 44 | | |
| | range | 4–19 | 6–22 | 23–96 | | |
| Light chains only | n | 1 | 4 | 4 | 6 | 0/9 (0) |
| Total No. | | 59 | 64 | 34 | 18 | 15/157 |

II, for patients within the same protein class (*e.g.* IgG) there was no significant correlation.

The relation of serum β2-m levels and creatinine levels in patients in whom renal impairment is indicated by elevated creatinine, is shown in Fig. 4, where comparison is made with the regression slopes for these variates in chronic renal failure and systemic lupus erythematosus, as published by Hall (1979). It will be seen that there is a wide range of serum β2-m levels for a particular creatinine level. The correlation coefficient, as measured by the Spearman ranking test, was $r = 0{\cdot}61$, $P = 0{\cdot}01$. In the longitudinal studies, levels of serum β2-m >15 mg/l were always associated with creatinine >140 μM. But considerable change in serum β2-m levels, either downwards during the reduction of the larger tumour burdens or upwards as the mass expanded, could occur without a coincidental change in serum creatinine.

FIG. 4.—Serum β2-m levels in patients with elevated serum creatinine. The regression line for systemic lupus erythematosus (SLE) and chronic renal failure are from Hall (1979).

### DISCUSSION

The discriminant power of serum β2-m level of 4 mg/l is apparent from the survival studies (Fig. 1) where good and bad prognostic groups are distinguished. It will require a larger series to define the level for optimal separation of these 2 groups. There is also a clear relation between serum β2-m levels and estimates of tumour mass, using the system devised by Durie & Salmon (Table I). On the other hand, the level of serum β2-m does not correlate with the amount of paraprotein in the blood, whatever its class. The serum β2-m level may follow the paraprotein level within a given patient, but this correlation is not seen when many patients are considered collectively, or when they are subdivided according to their paraprotein class.

At present it is uncertain what is the source of the raised serum β2-m in myelomatosis where frank renal failure has not supervened. Excessive production and abnormal β2-m elimination by the kidney could both be contributory factors. Once the creatinine begins to rise, the effect of hyperproduction is magnified and the serum β2-m is elevated more than is usual for chronic renal disease. Assuming that the raised levels are due to excess production, it is not known whether this β2-m comes from the mature plasma cells, their precursors or other cells of the lymphoid series. Studies of lymphoid disease indicate that raised serum β2-m can accompany a variety of benign and malignant diseases involving B or T lymphocytes (Cooper & Späti, 1979). Whatever the cell type or types contributing to the production of β2-m, its origin is most probably from the turnover of HLA on cell membranes, where β2-m forms the light chain of HLA (Cresswell *et al.*, 1974).

In practice the measurement of serum β2-m, at first presentation, would appear to be useful for the stratification of multiple myeloma, especially when the creatinine level is normal. The frequency of a renal impairment at first presentation has been reported as 43/237 (18·1%) by Woodruff *et al.* (1979), and 18% by Alexanian *et al.* (1975) using criteria less strict than ours.

High levels of serum β2-m at diagnosis or after 12–18 months carry a poor prognosis, and such levels are frequently encountered in IgA and IgG myeloma without an elevated serum creatinine (Table II). Furthermore, serum β2-m could well be a better reflection of the tumour mass than the serum paraprotein levels, and may offer a particular advantage for the monitoring of Bence Jones myelomatosis, as it is difficult to measure the serum concentrations of light chains accurately.

Severe acute infections do not necessarily raise serum β2-m, which is independent of the serum acute-phase reactive protein response (Cooper & Späti, 1979). This may account for the lack of rise of β2-m in the terminal periods of life of those patients in Fig. 1 and 2 whose intercurrent infection was the immediate cause of death.

Measurement of serum β2-m is simpler and less problematical than existing staging systems and paraprotein estimations. It may prove particularly applicable to the stratification and subsequent monitoring of patients with myelomatosis in the multi-centre clinical trial.

S.K. is supported by the Leeds United Hospitals Special Trustees. We are grateful to Dr B. Späti and Dr B. E. Roberts for their help in this study.

### REFERENCES

ALEXANIAN, R., BALCERZAK, S., BONNET, J. D. & 4 others (1975) Prognostic factors in multiple myeloma. *Cancer*, **36**, 1192.

BELLEVILLE, F., BERTRAND, F. & NABET, P. (1978) Beta 2-microglobulin and monoclonal gammopathies. *Pathol. Biol.*, **26**, 348.

CHRONIC LEUKEMIA–MYELOMA TASK FORCE (1973) Proposed guidelines for protocol studies II. Plasma cell myeloma. *Cancer Chemother. Rep.*, **4**, 145.

COOPER, E. H. & SPÄTI, B. (1979) The biochemical monitoring of lymphoproliferative disease. In *Tumour Markers: Impact & Prospects*. Eds Boelsma & Rümke. Elsevier/North-Holland Biomedical Press. p. 243.

CRESSWELL, P., SPRINGER, T., STROMINGER, J. L., TURNER, M. J., GREY, H. M. & KULO, R. T. (1974) Immunological identity of the small subunit of HLA antigens and β2-microglobulin and its turnover on the cell membrane. *Proc. Natl Acad. Sci. U.S.A.*, **71**, 2123.

DEFRONZO, R. A., COOKE, R., WRIGHT, J. R. & HUMPHREY, R. L. (1978) Renal function in patients with multiple myeloma. *Medicine*, **57**, 151.

DURIE, B. G. M. & SALMON, S. E. (1975) A clinical staging system for multiple myeloma. *Cancer*, **36**, 842.

HALL, P. W. (1979) β2-microglobulin in renal disease. In *Phadedoc Diagnostic Communications* Uppsala: Pharmacia. p. 36.

KAPLAN, E. L. & MEIER, P. (1958) Non-parametric estimation from incomplete observations. *J. Am. Statist. Ass.*, **53**, 457.

PETO, R., PIKE, M. C., ARMITAGE, P. & 7 others (1977) Design and analysis of randomized clinical trials requiring prolonged observation of each patient. II Analyses and examples. *Br. J. Cancer*, **35**, 1.

REVILLARD, J. P. (1979) La beta 2 microglobuline: Structure, fonction et métabolisme. *Lyon Médical*, **241**, 681.

SHUSTER, J., GOLD, P. & POULIK, M. D. (1976) Beta 2-microglobulin levels in cancerous and other disease states. *Clin. Chim. Acta*, **67**, 307.

WOODRUFF, R. K., WADSWORTH, J., MALPAS, J. S. & TOBIAS, J. S. (1979) Clinical staging in multiple myelomatosis. *Br. J. Haematol.*, **42**, 199.